# The Herbal Formula CWBSD Improves Sleep Quality Dependent on Oral Microbial Type and Tongue Diagnostic Features in Insomnia

**DOI:** 10.3390/jpm11050325

**Published:** 2021-04-21

**Authors:** Min-Jee Kim, Shambhunath Bose, Na-Rae Shin, Seohyun Park, Ojin Kwon, Eun-Ji Song, Young-Do Nam, Byung-Soo Koo, Dong-Hyun Nam, Jun-Hwan Lee, Hojun Kim

**Affiliations:** 1 Department of Rehabilitation Medicine of Korean Medicine, Dongguk University, Goyang 10326, Korea; eunmin23@naver.com (M.-J.K.); bose.sn@gmail.com (S.B.); sskr1207@nate.com (N.-R.S.); barksh84@naver.com (S.P.); 2 Division of Clinical Medicine, Korea Institute of Oriental Medicine, Daejeon 34054, Korea; cheda1334@kiom.re.kr; 3 Research Group of Healthcare, Korea Food Research Institute, Wanju-gun 55365, Korea; songej486@hanmail.net (E.-J.S.); youngdo98@kfri.re.kr (Y.-D.N.); 4 Department of Food Biotechnology, Korea University of Science and Technology, Daejeon 34113, Korea; 5 Department of Korean Neuropsychiatry, Dongguk University, Goyang 410-773, Korea; koobs@dongguk.ac.kr; 6 Department of Biofunctional Medicine and Diagnosis, College of Korean Medicine, Sangji University, Wonju 26382, Korea

**Keywords:** Cheonwangbosim-dan, insomnia, tongue diagnosis, oral microbiota, clinical trial

## Abstract

Cheonwangbosim-dan (CWBSD) is a traditional Korean herb formula that has been widely prescribed for insomnia patients with a heart-yin deficiency (HYD) pattern. Several studies have reported that heart function and insomnia are interrelated, and few have explored associations between insomnia, oral microbiota, and tongue diagnosis. This study aimed to evaluate the effects of CWBSD on primary insomnia, tongue diagnosis, and oral microbiota. At baseline, 56 patients with primary insomnia were assigned to two groups, a HYD group and a non-HYD (NHYD) group and they took CWBSD for 6 weeks. During the study, Pittsburgh Sleep Quality Indices (PSQIs) and Insomnia Severity Indices (ISIs) decreased significantly in both groups. However, the PSQI reduction observed in the HYD group was greater than in the NHYD group and sleep times increased only in the HYD group. As sleep quality improved, the amount of tongue coating increased at the posterior tongue, where heart function appears. At baseline, the HYD and NHYD group had a specific oral microbiota (*Veillonella* at genus level), but no significant change was observed after taking CWBSD. Additionally, subjects were divided into two oral microbiota types (“orotypes”). The genera *Prevotella*, *Veillonella*, or *Neisseria* were abundant in each orotype. The reduction in PSQI in orotype 1 during the 6-week treatment period was greater than in orotype 2. In conclusion, this study shows that CWBSD could be used to treat primary insomnia in patients with a HYD pattern as determined using tongue diagnosis and oral microbiota distributional patterns.

## 1. Introduction

Insomnia refers to difficulties initiating or maintaining sleep and causes discomfort, loss of energy, fatigue, mood disturbances, etc., which ultimately significantly impair normal daytime activities [1,2]. Insomnia can be divided into two subtypes: primary and secondary insomnia [1]. In primary insomnia, the sleeping disorder is not directly associated with any other health condition or problem, while in secondary insomnia, the sleeping problem is linked to a health condition such as asthma, depression, arthritis, cancer, or heartburn. Although insomnia is considered to be a common neuropsychiatric disorder with a prevalence of 10–30% worldwide [3,4,5], the exact pathophysiology of this sleeping disorder is poorly understood [6].

Insomnia has been reported to be linked with cardiovascular diseases due to involvement of the sympathetic nervous system (SNS) and hypothalamic-pituitary-adrenal activity [7,8]. The SNS along with the parasympathetic nervous system constitutes the autonomic nervous system (ANS), which plays a crucial role in the physiology and pathology of cardiovascular diseases. Therefore, regulation of ANS activity is an important aspect of the treatment and management of cardiac dysfunction resulting from autonomic imbalance [9]. In traditional Korean medicine (TKM), heart-yin deficiency (HYD) is one of the most common etiologies of insomnia and is a pathological condition characterized by anxiety, tidal fever, night sweats, facial flushing, a thready, rapid pulse, a dry mouth, a red tongue, and less tongue coating [10]. Therefore, in both modern physiology and TKM, insomnia and heart function are closely related.

A number of studies have been conducted to understand the relationship between heart function and oral health. For instance, bacteremia caused by oral inflammation or tooth extraction can stimulate autonomic nerves and cause atrial fibrillation as well as infective endocarditis [11,12]. Additionally, antiseptic mouthwashes impair the oral bacteria-mediated conversion of nitrate to nitrite, which eventually increases blood pressure as nitrite is a potential vasodilator [13,14]. Furthermore, links between oral microbiota and heart diseases, especially coronary artery disease, have been reported. For example, five oral commensal bacteria (*Campylobacter rectus*, *P. gingivalis*, *Porphyromonas endodontalis*, *P. intermedia*, and *Prevotella nigrescens*) were found to be specific for coronary artery disease as compared with several non-cardiac disorders [15].

The evaluation of tongue characteristics is an important diagnostic method practiced in TKM to investigate physiological function and pathological changes, especially those of the heart and spleen [16,17]. However, as the conventional diagnostic method of simply observing the tongue is restricted by several external and subjective factors, modern tongue-based diagnostic systems (TDSs) have been developed to make tongue examination more objective [6,16]. In a previous clinical trial on 60 patients with functional dyspepsia, tongue coating percentage, as assessed by TDS, was significantly related with tongue coating weight, and tongue diagnoses by TDS and traditional medicine experts exhibited high agreement [18]. Additionally, an earlier clinical computerized TDS study revealed that patients with a yin-deficient pattern tended to have a redder tongue and a thinner tongue coating than non yin-deficient patients [19]. Researchers also recently reported that next-generation sequencing might be useful for investigating the relation between tongue diagnosis and oral microbiota. In a previous clinical study, tongue coating microbiota associated with cold/hot syndromes were recognized in patients with chronic atrophic gastritis and found to have potential as novel holistic biomarkers for characterizing patient subtypes [20]. In another clinical trial, Bacillus was significantly observed only in chronic erosive gastritis (CEG) patients with a yellow tongue coating but not in healthy volunteers with a thin white tongue coating or CEG patients treated with a Ban Xia Xie Xin decoction [21].

Cheonwangbosim-dan (CWBSD) is a traditional Korean herbal formula widely prescribed for insomnia patients, especially those with a HYD pattern, due to its efficacy, few side effects, and low cost [22,23]. However, only a small number of studies have investigated the mode of action of CWBSD in insomnia [24]. Moreover, it is difficult to objectively identify patients with this pattern suitable for treatment with CWBSD. Indeed, to the best of our knowledge, no study has been conducted to address the symptoms and prognosis of insomnia in terms of cardiac and autonomic nerve functions, tongue diagnosis, and oral microbiota composition, and no report has been issued on the effect of CWBSD on insomnia with respect to these three aspects. Accordingly, we investigated the impact of CWBSD on the sleep quality of insomnia patients with respect to tongue diagnoses and oral microbial compositions and sought to develop a method that predicts drug responsiveness to CWBSD.

## 2. Materials and Methods

### 2.1. Study Design

This study was conducted using a prospective, open-label, single-center clinical trial design and was conducted between May 2018 and February 2019 at Ilsan Dongguk University Hospital, Korea (Trial Registration Number: KCT0003668: https://cris.nih.go.kr/cris/index.jsp, accessed on 17 April 2021). Patients took one pouch of CWBSD (20 small pellets/3.75 g/pouch) one hour after dinner for 6 weeks. Vital signs, anthropometric parameters, sleep qualities, and cardiac and autonomic nerve functions were measured at baseline and 3 and 6 weeks after study commencement. Laboratory parameters, tongue features, and oral microbial compositions were analyzed at baseline and study termination (after 6 weeks of treatment). In addition to being assessed at baseline and after 3 and 6 of treatment, Pittsburgh Sleep Quality Indices (PSQIs) and Insomnia Severity Indices (ISIs) were also measured 4 weeks after treatment completion (week 10) by conducting standardized telephonic interviews with patients. The study was conducted in accordance with the Declaration of Helsinki [25] and the study protocol was approved by the Institutional Review Board of Ilsan Dongguk University Hospital (No: DUIOH 2018-02-001-003). Signed informed consent was obtained from all patients before they were enrolled in the study.

### 2.2. Medication

The small, dark brown CWBSD pellets used were produced by Jungwoo Newpharm Co. (Seoul, Korea), which has been awarded a certificate for Good Manufacturing Practice (GMP) by the Korean Ministry of Food and Drug Safety. The composition of the formulation is shown in Table 1. At the end of study week 6, subjects returned any remaining CWBSD. Overall drug compliance during the study was in excess of 80%. When individual drug compliance was less than 80%, the subject concerned was considered to have poor compliance and excluded from analysis, per protocol.

### 2.3. Subjects

Male and female South Korean adults, aged 19–65 years, with a diagnosis of primary insomnia, were included in this study. The study inclusion criteria were: (1) experience of insomnia > once a week for the previous 3 months; (2) baseline PSQI and ISI scores of at least 6 and 8 points, respectively; and (3) a Beck Depression Index (BDI) score of < 24 points (to exclude subjects whose sleep disorders were caused by depression). Those that had suffered from acute insomnia within the previous 2 weeks were excluded, as were subjects with a history of heart disease (cardiac failure, angina pectoris, myocardial infarction, or ischemic cardiac arrest); a medical condition known to induce insomnia (organic brain disorder such as stroke and encephalitis, hypothyroidism, hypoglycemia, disorders of liver or kidney, or chronic respiratory disease); or a history of or a current mental disorder (schizophrenia, bipolar disorder, severe depression, etc.). In addition, subjects were excluded if they had a condition that might affect sleep (e.g., pain) or worked night shifts, if they had taken insomnia-related drugs, probiotics, or antibiotics, or participated in another clinical trial during the month before study commencement. Subjects with a dental prosthesis and pregnant or breastfeeding women were also eliminated. During the study, volunteers wore an actigraph and were instructed to avoid caffeine after 2 p.m., but were allowed one alcoholic drink after 6 p.m. All eligible subjects agreed to comply with the study protocol, voluntarily signed an informed consent form, and received CWBSD treatment for 6 weeks. Subjects were also required to maintain their usual diet and physical activities during the study period.

### 2.4. Identification of the TKM Pattern

To identify the TKM pattern of insomnia, we chose the Pattern Identification Tool-Insomnia (PIT-Insomnia) tool developed by seventeen South Korean specialist experts in the field of Korean medicine neuropsychiatry. PIT-Insomnia analysis was based on the responses of subjects in a self-report form to 47 questions developed from published Korean and Chinese literature. The questions were linked to various symptoms related to the autonomic nervous system, sleep patterns, cognitive and emotional functions, the digestive system, the urinary and urogenital systems, and others [26]. According to their responses, we categorized subjects into two groups, a HYD (heart-yin) group and a non-HYD (NHYD) group. Notably, a clinical study conducted on 38 insomnia patients to assess the reliability and validity of PIT-Insomnia analysis and to verify correlations between this tool and psychological tests [27] showed that PIT-Insomnia scores had moderate reliability and showed some concurrent validity with the PSQI, an indicator of insomnia severity. In addition, PTI-Insomnia scores showed some correlations with specific psychological tests, such as the Korean-Beck Depression Inventory, the Korean Adaptation of Spielberger’s STAI, and the Korean version of the Symptom Checklist-95-Revision test.

### 2.5. Analysis of Sleep Patterns

Sleep patterns of participants were analyzed based on responses to two types of questionnaires and actigraph data. We assessed sleep quality and insomnia severity using PSQI and ISI questionnaires. The PSQI questionnaire consists of 19 questions that assess sleep quality and patterns over the previous month. The sum of the 7 sleep-related components of this questionnaire yields global scores that range from 0 to 21 points. A total score of greater than 5 points indicates poor sleep quality. On the other hand, the ISI contains 7 questions that evaluate the severity of insomnia over the previous month. Item responses are rated using a 5-point Likert scale, and thus, total scores range from 0 to 28 points. A total score of over 8 points suggests insomnia, and a score of ≥22 points indicates severe insomnia [28]. Subjects also maintained a daily sleep diary, and this information was used to analyze actigraph data.

Actigraphy was conducted using a wrist-worn mini motionlogger (Ambulatory Monitoring Inc., Ardsley, New York, NY, USA). Subjects wore actigraphs continuously on the non-dominant wrist for two weeks: for one week prior to CWBSD administration and during the last (6th) week of the study, except when bathing or taking a shower. Actigraphs were initialized under sleep-wake zero-crossing mode, with a fixed epoch of 1 min, and an amplifier setting of 18. Subjects were instructed to press the event-mark on the side of the actigraph before they fell asleep and again after waking up.

### 2.6. Analyses of Cardiac Function and Autonomic Nerve Function

After 0, 3, or 6 weeks of treatment, cardiac and autonomic nerve functions were assessed by measuring heart rate variabilities (HRV) and pulse rates for 3 min after a 5 min rest period using an SA-6000 autonomic function test and blood circulation assessment device (Medicore Co. Ltd., Seoul, Korea). This device functions as a photoplethysmometer that measures arterial waves propagated by changes in peripheral blood volume. Signals were captured using a photosensor worn by subjects on a finger. HRVs were measured after modifying instantaneous heart rates from pulse waves and then analyzed. Using this data, autonomic nervous system function, low frequency power in normalized units (LFn), and high frequency power in normalized units (HFn) were evaluated.

### 2.7. Analysis of Tongue Features

After waking up, subjects visited our hospital in at least a 4 h fasted state or tooth brushing. Images of the tongue dorsum were captured using a digital camera using manually adjusted white balance settings and subsequently analyzed using a CTS-1000 computerized tongue image analysis system (Daiseung Medics, Co., Seoul, Korea). Coated tongue areas were distinguished based on red saturation differences and the mean color values of tongue bodies and coatings were analyzed. Whole tongue regions were then divided into six subregions as performed for Winkel Tongue Coating Index (WTCI) determination [29]. The number of pixels in each of these subregions was counted, and ratios of tongue coating to tongue body areas were calculated. Details of the color correction and segmentation processes used have been previously described [16,19]. Finally, tongue coating samples were collected from the surface of the tongue dorsum by swabbing with a sterile spatula from the tongue root to tip for oral microbial analysis.

### 2.8. Oral Microbial Analysis

Fresh tongue coating samples were thoroughly mixed with saline and centrifuged. The resultant supernatants were collected and stored immediately at −80 °C for microbiome study. Metagenomic DNA was isolated using a DNA Miniprep kit (QIAGEN, Hilden, Germany) according to the manufacturer’s instructions. The V1–V2 region of the 16S rRNA gene was amplified using a Thermal Cycler PCR system (BioRad, Hercules, CA, USA), after which amplicons were purified using a LaboPass PCR purification kit (COSMO GENTECH, Seoul, Korea). Each sample was amplified with a barcoded primer so that individual reads could be identified and sorted before analyzing multiplex amplicon sequencing data. Equimolar concentrations of amplicons from different samples were pooled in equal proportions. Sequencing libraries were constructed from pooled amplicon samples using the Ion Xpress Plus fragment library kit (Thermo Fisher Scientific, Waltham, MA, USA). The quality and quantity of each constructed library was verified using a BioAnalyzer 2100 microfluidics-based device (Agilent, Santa Clara, CA, USA) using a High Sensitivity DNA kit (Agilent, Santa Clara, CA, USA). Sequencing reactions were performed using an Ion Torrent Personal Genome Machine system (Thermo Fisher Scientific, Waltham, MA, USA).

The selection of operational taxonomic units (OTUs; 97% identity; Greengenes database: http://greengenes.lbl.gov, accessed on 17 April 2021), taxonomic assignments, and phylogenetic reconstructions were carried out using the Quantitative Insights into Microbial Ecology (QIIME) software package (Version 1.9.1, University of Colorado, Boulder, CO, USA). Taxa with significantly different abundances in the HYD and NHTD groups were identified using the linear discriminant analysis effect size (LEfSe; Hutlab, Boston, MA, USA) tool using a web-based program (http://huttenhower.sph.harvard.edu/galaxy accessed on 17 April 2021) and visualized using Graphpad Prism 5 (GraphPad, San Diego, CA, USA). During this analysis, the alpha value of the factorial Kruskal–Wallis test among classes was set to <0.05 and the threshold of the logarithmic linear discriminant analysis (LDA) score was set to >2.0. Alpha diversities were measured using Shannon’s and Simpson’s indices, and beta diversities were determined using Bray–Curtis/unweighted UniFrac/weighted UniFrac distance. Finally, using these data, non-metric multidimensional scaling (NMDS)/principal coordinate analysis (PCoA) plots were constructed with the help of QIIME. Additionally, we analyzed oral microbial types (hereafter referred to as “orotypes”) at the genus level using the R code provided by MetaHIT (https://enterotype.embl.de/enterotypes.html accessed on 17 April 2021). Samples were clustered using the partitioning around medoids clustering algorithm [30], and an optimal number of clusters was validated using the Calinski–Harabasz index [31]. Sample distributions were calculated based on relative genus abundances using the Jensen–Shannon divergence (JSD) distance metric and visualized using PCoA plots. The Insomnia_orotype_input.csv file was used as raw data for orotype analysis.

### 2.9. Analyses of Blood Parameters

Routine blood tests were performed at baseline and at the termination of CWBSD treatment at the end of week 6. Levels of white blood cells (WBC), red blood cells (RBC), hemoglobin (Hb), hematocrit (Hct), platelets (PLT), neutrophils (NEU), and lymphocytes (LYM) were measured using a Sysmex XN 9000 hematology analyzer (Sysmex Inc., Kobe, Japan).

### 2.10. Statistical Analysis

In general, continuous data are presented as means and 95% confidence intervals and were analyzed using the independent two-sample *t*-test or Wilcoxon’s rank-sum test. Categorical data are presented as frequencies and percentages (%) and were analyzed using the Chi-square test or Fisher’s exact test. In most cases, statistical analysis was performed using the two-tailed test method. The statistical evaluation of the effectiveness of CWBSD on total sleep time (TST) as a primary endpoint was performed using the paired *t*-test or Wilcoxon’s signed-rank test, depending on the normality of data. Statistical analysis of secondary endpoints including sleep questionnaire (PSQI and ISI) results, cardiac function, autonomic nerve function, tongue features, and oral microbial population were performed in the same way as the evaluation of the primary endpoint. For secondary endpoints, categorical variables were analyzed using the Chi-square test or Fisher’s exact test. More specifically, the above-mentioned primary and secondary endpoints were statistically evaluated using analysis of covariance with groups as fixed factors and baseline measurements as covariates. Additionally, Pearson’s or Spearman’s correlation tests were used to investigate correlations between cardiac function, oral microbiota, and tongue features. Fisher’s discriminant analysis was used to develop a prediction equation for CWBSD responsiveness. A decrease in ISI score by >30% from baseline was considered to indicate responsiveness to CWBSD. Several oral microbiotas at the genus level were considered independent variables. Statistical analysis was conducted using SPSS version 20.0 (SPSS Inc., Chicago, IL, USA), and statistical significance was accepted for *p* values < 0.05.

## 3. Results

### 3.1. Baseline Characteristics of the Subjects and Adverse Events

**Figure 1:**
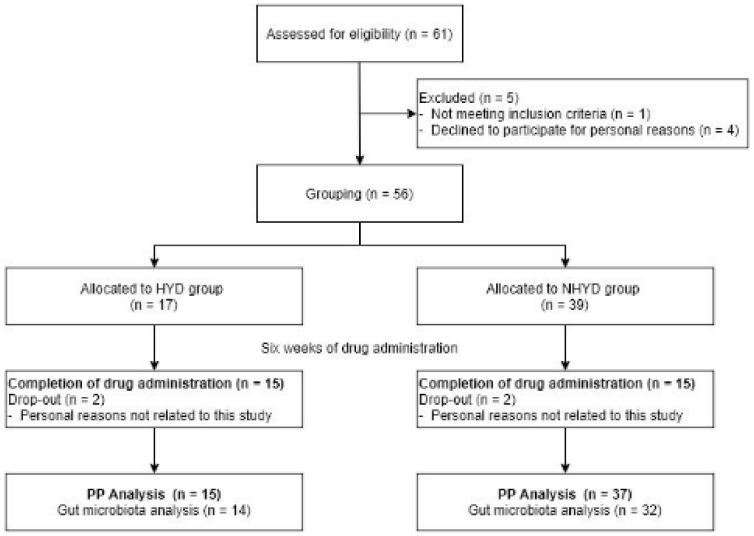
Study flow chart of patient progress through the clinical trial from enrollment to analysis.

Initially, 61 subjects were screened for participation in this study, but 5 were excluded; 1 for not fulfilling the inclusion criteria and 4 for personal reasons. Accordingly, a total of 56 subjects were enrolled in this trial. The 56 subjects were subsequently divided into two groups; 17 were assigned to the HYD group and 39 to the NHYD group, depending on their TKM insomnia patterns. During the study period, 2 subjects from each group dropped out for personal reasons which were not related to this study. Therefore, 15 of the 17 subjects (88.24%) in the HYD group and 37 of the 39 subjects (94.87%) in the NHYD group completed the study. However, we could not perform oral microbial analyses on 6 subjects who completed the trial because of limited tongue coating samples (Figure 1). At baseline, subjects in the HYD group had significantly higher BDI and ISI scores than those in the NHYD group (Table 2). In addition, the HYD group had higher PSQI scores and pulse rates, although these differences were not statistically significant. No adverse event was reported by any participant during the study period.

### 3.2. CWBSD Treatment Improved Sleep Quality Objectively and Subjectively

To analyze the subjective pattern and quality of sleep, the participants completed two questionnaires, PSQI and ISI. The HYD and NHYD groups both showed a significant decline in ISI and PSQI scores at the end of the treatment period and at follow-up on week 10. More specifically, ISI scores fell by an average of 8.33 points in the HYD group (*p* < 0.001) and 6.47 points in the NHYD group (*p* < 0.001) in response to treatment at 6 weeks. On the other hand, the HYD group had a significantly lower PSQI score (*p* < 0.05) than the NHYD group at this time. More specifically, the reduction in PSQI score from baseline was approximately two times greater in the HYD group (mean score reductions were 6.00 and 3.53).

**Figure 2:**
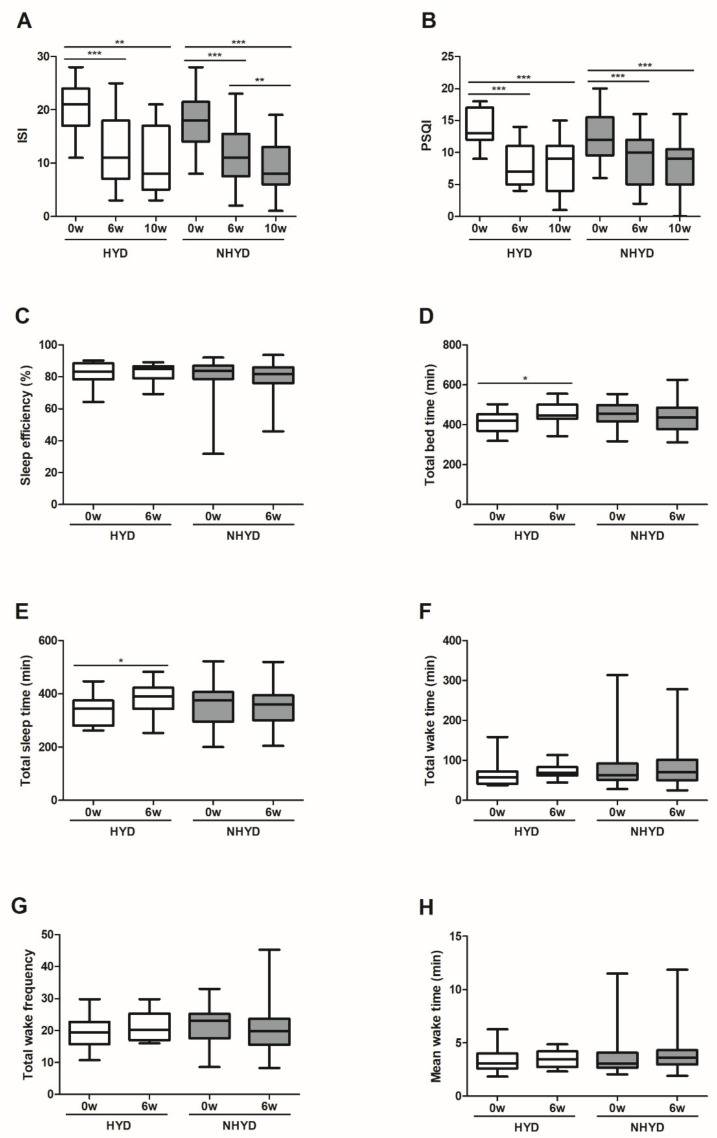
Effects of CWBSD on sleep quality. Changes in (**A**) ISI, (**B**) PSQI, (**C**) sleep efficiency, (**D**) total bedtime, (**E**) total sleep time, (**F**) total wake time, (**G**) total wake frequency, and (**H**) mean wake time after 6 weeks of CWBSD administration. The lines, boxes, and whiskers in the box plots represent the median (50th percentile), 25th and 75th percentiles, and the minimum-to-maximum distribution of replicate values, respectively (* *p* < 0.05, ** *p* < 0.01, *** *p* < 0.001). ISI: Insomnia Severity Index; PSQI: Pittsburgh Sleep Quality Index; CWBSD: Cheonwangbosim-dan.

Subjects were also instructed to wear actigraphs to monitor sleep patterns and quality. A significant increase in total bedtime (TBT) and TST (*p* < 0.05) were seen in the HYD group, but not in the NHYD group, in response to treatment with CWBSD for 6 weeks. In keeping with these observations, increases in TBTs and TSTs from baseline were significantly higher in the HYD group (*p* < 0.01) (Figure 2).

### 3.3. CWBSD Treatment Changed Tongue Coating Distribution, Especially in the HYD Group

**Figure 3:**
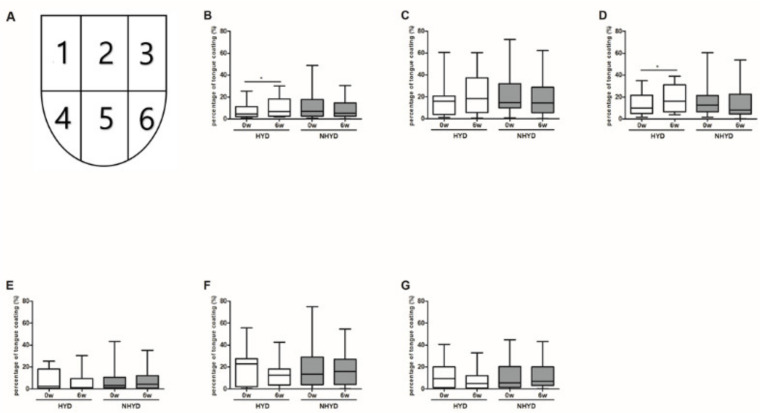
Effects of CWBSD on Winkel Tongue Coating Indices. Changes in the percentage of tongue coating as quantified by Winkel Tongue Coating Indices after 6 weeks of CWBSD treatment. (**A**) Winkel Tongue Coating Index is shown as a picture. Graphs represent tongue coating percentages (PTC, %) of (**B**) 1, (**C**) 2, (**D**) 3, (**E**) 4, (**F**) 5, and (**G**) 6 areas divided by Winkel Tongue Coating Indices. The lines, boxes, and whiskers in the box plots represent the median (50th percentile), 25th and 75th percentiles, and the minimum-to-maximum distribution of replicate values, respectively (* *p* < 0.05). CWBSD: Cheonwangbosim-dan.

Tongue coating distributions were evaluated using WTCIs. Significant increases in tongue coating in areas 1 and 3 were observed in the HYD group (*p* < 0.05), but not in the NHYD group, after treatment for 6 weeks (Figure 3). Although changes in mean blue color values of tongue bodies due to CWBSD treatment were significantly different in the two groups (*p* = 0.031), pixel counts of whole tongue areas were significantly decreased in the HYD group on week 6 (*p* = 0.026) (Table S1), but not in the NHYD group. These two parameters were not clinically important as far as the objective of the study is concerned.

### 3.4. Oral Microbiota Patterns at Baseline and Changes in Oral Microbial Compositions after CWBSD Administration

**Figure 4:**
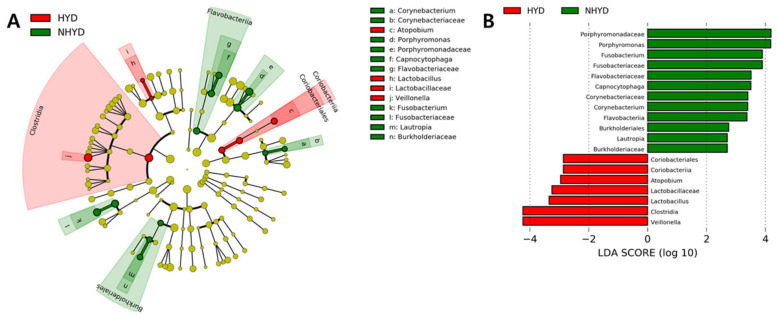
Oral microbiota differences between groups. (**A**) Taxonomic representation of statistically and biologically consistent differences between the HYD and NHYD groups. Differences are represented by the color of the most abundant class (red indicating HYD, green representing NHYD, and yellow meaning non-significant). Circle diameters are proportional to taxon abundances. (**B**) Histogram of LDA scores for differential abundances. The cladogram was calculated by LEfSe and displayed according to effect size.

Data from 16S rRNA sequencing revealed that alpha and beta diversities of oral microbial populations in the NHYD and HYD groups did not differ significantly after CWBSD treatment (Figure S1). On the other hand, cladograms and LDA scores, the two metrics required for LEfSe assessment, indicated that at baseline, *Corynebacterium*, *Porphyromonas*, *Capnocytophaga*, and *Fusobacterium* genera were more abundant in the NHYD group than in the HYD group (Figure 4), and that *Atopobium*, *Lactobacillus*, and *Veillonella* genera were more abundant in the HYD group. 

**Figure 5:**
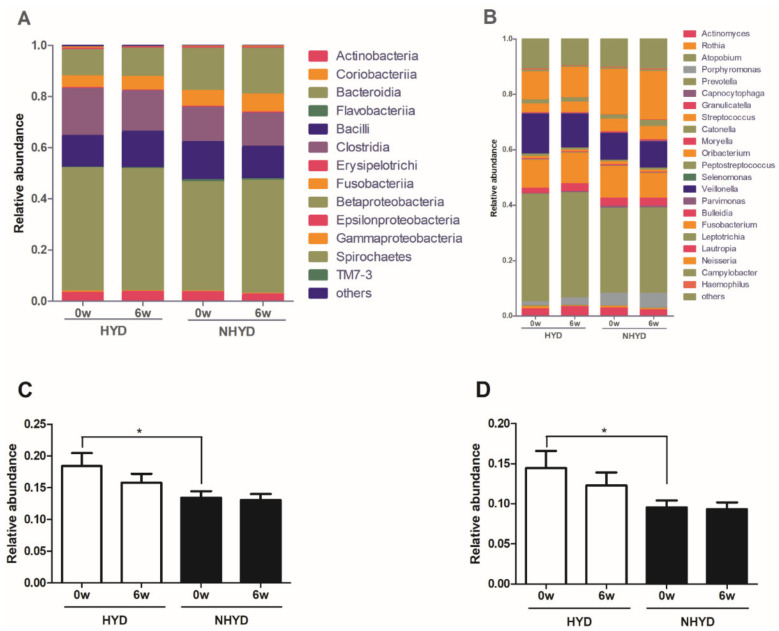
Overall effects of CWBSD on the relative abundances of oral microbiota. Overall compositional changes of oral microbiota are demonstrated in (**A**,**B**), and changes in the relative abundances of individual oral microbiota in (**C**,**D**). (**A**) Relative abundances at the class level. (**B**) Relative abundances at the genus level. (**C**) Relative abundances of Clostridia at the class level. (**D**) Relative abundance of *Veillonella* at the genus level. Data are presented as means ± standard deviations (* *p* < 0.05). CWBSD: Cheonwangbosim-dan.

Further analysis of 16S rRNA sequencing data showed that at the class level, *Bacteroidia*, *Clostridia*, *Bacilli*, *Betaproteobacteria*, and *Fusobacteriia* were the most abundant oral microbiota in the NHYD and HYD groups at baseline, while at the genus level, *Prevotella*, *Veillonella*, *Streptococcus*, and *Neisseria* were the most abundant oral bacteria in both groups at baseline. However, at the class and genus levels, oral microbial populations were significantly altered by treatment in both groups. Nevertheless, we found that at baseline, the abundances of *Clostridia* at the class level and of *Veillonella* at the genus level were significantly higher (*p* < 0.05) in the HYD group (Figure 5).

### 3.5. Correlation between Clinical Parameters and Oral Microbiota Distributions

**Figure 6:**
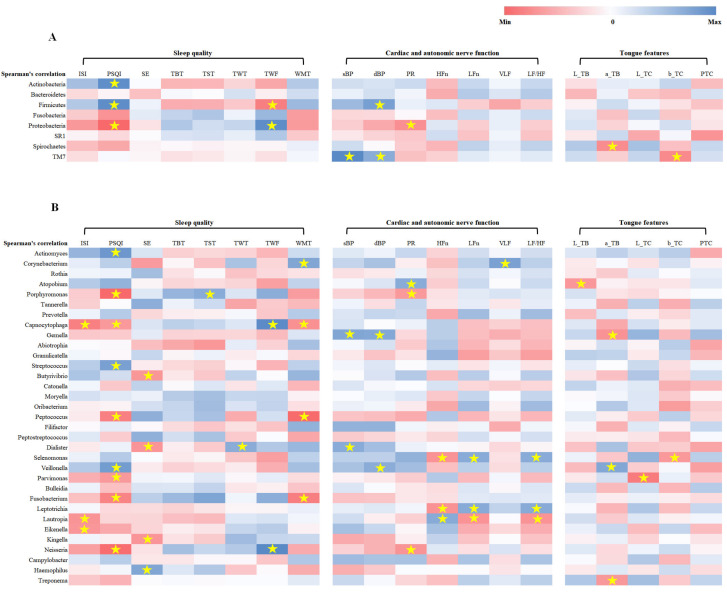
Correlation analysis between clinical parameters and oral microbiota. (**A**) Correlation analysis between clinical parameters and the eight most abundant phylum-level microbiota. (**B**) Correlation analysis between clinical parameters and the 34 most abundant microbiota at the genus level. Correlation coefficients were calculated by Spearman’s correlation analysis. * *p* < 0.05. ISI: Insomnia Severity Index; PSQI: Pittsburgh sleep quality index; SE: sleep efficiency; TBT: total bedtime; TST: total sleep time; TWT: total wake time; TWF: total wake frequency; WMT: mean wake time; SBP: systolic blood pressure; DBP: diastolic blood pressure; PR: pulse rate; HFn: high frequency power in normalized units; LFn: low frequency power in normalized units; VLF: very low frequency; LF/HF ratio: low frequency/high frequency ratio; L_TB: mean CIE-lightness value of pixels in the tongue body area; a_TB: mean CIE-red saturation value of pixels in the tongue body area; L_TC: mean CIE-lightness value of pixels in the tongue coating area; b_TC: mean CIE-yellow saturation value of pixels in tongue coating area; PTC: percentage of tongue coating area versus whole tongue area.

Spearman’s correlation analysis of the 16S rRNA sequencing data of oral microbiota revealed that at baseline of the 8 most abundant microbiomes at the phylum level in all subjects, *Firmicutes* was positively correlated with PSQI and diastolic BP and negatively correlated with TWF, while *Proteobacteria* was positively correlated with TWF and negatively correlated with PSQI and pulse rate (Figure 6A). *TM7* abundance was positively correlated with systolic and diastolic BPs, but negatively correlated with CIE-b* values (a measure of yellowness) of tongue coatings. Of the top 34 most abundant oral microbiota at the genus level, *Veillonella* and *Streptococcus* abundances were positively correlated with PSQIs, while *Neisseria* showed a negative correlation (Figure 6B). Additionally, *Veillonella* abundance was positively correlated with diastolic BP and CIE-a*. On the other hand, *Peptococcus* and *Fusobacterium* abundances were negatively correlated with PSQI and mean wake time (WMT). *Selenomonas* and *Leptotrichia* were both negatively correlated with HFn and positively correlated with LFn, the two indices of the autonomic nervous system. In contrast, *Lautropia* abundance exhibited a positive correlation with HFn and a negative correlation with LFn and ISI. *Porphyromonas*, on the other hand, showed a negative correlation with PSQI and pulse rate and a positive correlation with TST. *Gemella* was positively correlated with systolic and diastolic BP and negatively correlated with CIE-a* values of tongue bodies.

### 3.6. CWBSD Did Not Affect Cardiac Function, Autonomic Nerve Function, or Blood Parameters

Cardiac function and autonomic nerve activity and blood parameters were relatively unaffected by CWBSD treatment in both of the study groups (Tables S2 and S3).

### 3.7. Sleep Quality at Baseline, CWBSD Responsiveness, and Microbial Compositions of Orotypes

**Figure 7:**
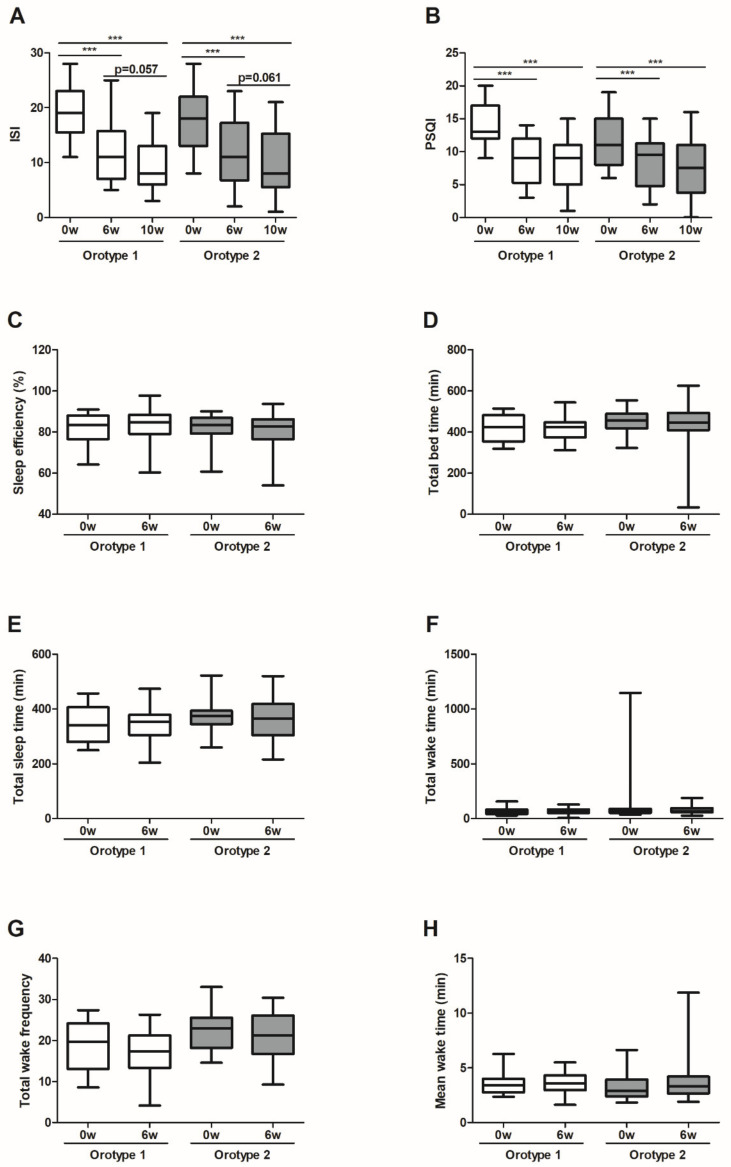
Effects of CWBSD on sleep quality by oral microbiota type. Changes in (**A**) ISI, (**B**) PSQI, (**C**) sleep efficiency, (**D**) total bedtime, (**E**) total sleep time, (**F**) total wake time, (**G**) total wake frequency, and (**H**) mean wake time after 6 weeks of CWBSD administration. The lines, boxes, and whiskers in the box plots represent the median (50th percentile), 25th and 75th percentiles, and the minimum-to-maximum distribution of replicate values, respectively (*** *p* < 0.001). ISI: Insomnia Severity Index; PSQI: Pittsburgh Sleep Quality Index; CWBSD: Cheonwangbosim-dan.

At baseline, PSQI score and pulse rate of the participants of orotype 1 were significantly higher than those of orotype 2 (Table 3). Interestingly, ISI and PSQI scores declined significantly among participants of orotype 1 and 2 over the 6-week treatment period and were sustained 4 weeks later. In particular, the amount of PSQI score decrease for orotype 1 (mean difference 5.55) was significantly greater (*p* = 0.045) than that for orotype 2 (mean difference 3.46) (Figure 7). However, subjects did not show significant intra- or inter-orotype differences with respect to tongue features, cardiac function, or autonomic nerve function in response to CWBSD treatment (Table S4).

**Figure 8:**
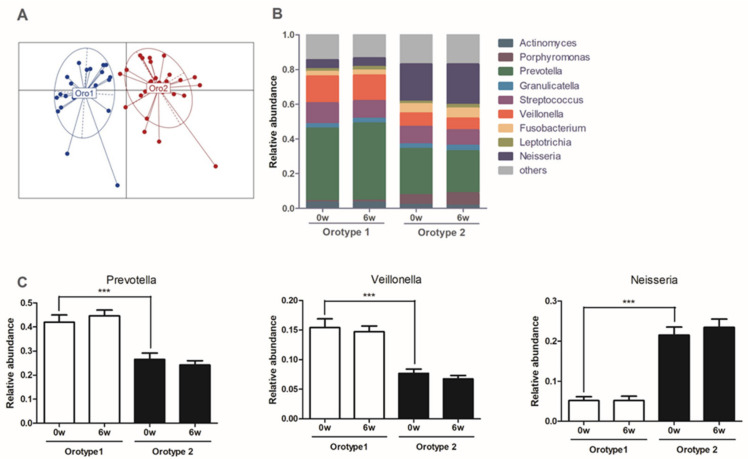
Identification of oral microbiota types. (**A**) Oral microbial communities in orotypes. (**B**) Relative abundances at the genus level for each orotype. (**C**) Relative abundances of the three bacterial taxa principally responsible for the dichotomization of orotypes. Data are presented as means ± standard deviations (*** *p* < 0.001).

Furthermore, no significant change in oral microbial composition was observed within or between orotype 1 and orotype 2 in response to CWBSD treatment (Figure 8). However, at baseline, the relative abundances of *Prevotella* and *Veillonella* taxa were significantly higher in the orotype 1 subgroup than in the orotype 2 subgroup, whereas the abundance of *Neisseria* taxon was significantly higher in the orotype 2 subgroup.

### 3.8. Equation Predicting CWBSD Response

Based on Fisher’s discriminant analysis of oral microbial data, *Paludibacter*, *Selenomonas*, *Peptococcus*, *Peptostreptococcus*, and *Gemella* were found to be candidate genera for predicting response to CWBSD treatment. A change in ISI score was regarded a measure of drug response. The equation obtained was: 

Estimated change of ISI = (21,781 × *Paludibacter* abundance) + (1306.0204 × *Selenomonas* abundance) + (2073.6681 × *Peptococcus* abundance) − (222.6118 × *Peptostreptococcus* abundance) + (2128.7933 × *Gemella* abundance)

Insomnia patients with an estimated discriminant analysis value of greater than 1.8525 would be regarded as CWBSD responders and those with a smaller value, non-responders. The sensitivity of this equation was 72.00% and its specificity 90.48%.

## 4. Discussion

In this study, we evaluated the impact of 6 weeks of CWBSD treatment on sleep quality, cardiac function, autonomic nerve function, tongue coating, and oral microbial composition in insomnia patients with two different TKM patterns, that is, HYD or NHYD. At baseline, BDI and ISI scores were significantly higher in the HYD group. Furthermore, PSQI score and pulse rate were non-significantly higher in the HYD group. It appears conceivable that insomnia patients with the HYD pattern are more depressed, have inferior sleep quality, and faster pulse rates than insomnia patients with the NHYD pattern. These findings are in keeping with previous reports that demonstrated in insomnia patients with a yin-deficiency pattern that PSQI scores and pulse rates were positively correlated with weighting of yin-deficiency [32,33]. 

After 6 weeks of CWBSD administration, sleep quality was improved according to subjective and objective indicators. At this time, PSQI and ISI scores were significantly lower than baseline levels in the HYD and NHYD groups. In particular, the magnitude of this fall in PSQI score in the HYD group was approximately twice that in the NHYD group. This is in agreement with a previous clinical study, in which exposure of insomnia patients with yin-deficiency and fire-hyperactivity syndrome to CWBSD for 4 weeks decreased PSQI scores and improved daytime functions, physical symptoms, and mood [34]. We also observed significant increases in TBT and TST in the HYD group, but not in the NHYD group, on week 6. These results indicate that CWBSD has the potential to improve both the subjective and objective aspects of sleep.

Although at baseline the patient in the HYD group tended to have redder tongues with less coating than the NHYD group, these differences were not significant. In parallel with a marked improvement in sleep quality, the amount of tongue coating was increased significantly in the HYD group in response to CWBSD treatment. We also noticed that this increase in tongue coating was more prominent on the posterior tongue. Since a small amount of coating is one of the main diagnostic features of the HYD pattern [19], it is conceivable that CWBSD can ameliorate the adverse impacts of HYD in insomnia patients.

Oral microbiota represent one of the most predominant microbial populations in the human body [35,36]. Since accumulating evidence indicates that oral microbiota play an important role in health [37,38,39], several studies have investigated the relationships between oral microbiomes and diseases, especially heart disease [11,12,13,14]. A recent report revealed that the diversity and abundance of oral bacteria differ significantly in healthy controls and chronic insomnia patients at multiple taxonomic levels [40]. Moreover, the diversity of oral and nasal microbiota increases in diseases such as severe obstructive sleep apnea and halitosis [41,42]. In our study, cladograms and LDA scores, the two major outputs of the LEfSe assessment, showed oral microbiome distribution patterns differed in the HYD and NHYD groups at baseline. More specifically, the NHYD group exhibited higher abundances of *Corynebacterium*, *Porphyromonas*, *Capnocytophaga*, and *Fusobacterium* genera, whereas the HYD group had higher abundances of *Atopobium*, *Lactobacillus*, and *Veillonella* genera. It has been reported that these four enriched microbiota in the NHYD group are related to oral diseases such as periodontal disease and tooth decay [43,44,45,46] and to heart infections. For instance, it has been reported that *Capnocytophaga haemolytica* can cause periodontal disease and aortic valve endocarditis [43,47]. Among the abundant oral microbiota in the HYD group, *Veillonella* is known to metabolize lactic acid [48], and *Lactobacillus* produces lactic acid, which can produce dental caries [49]. Furthermore, it has been shown that *Veillonella* is enriched in the nasal microbial populations of patients suffering from severe obstructive sleep apnea [41]. In our study, *Veillonella* was the only oral microbiota that demonstrated significantly higher abundance in the HYD group than in the NHYD group at baseline. Our study further revealed that diversity, as well as the composition of oral microbiota in both groups, did not change after the administration of CWBSD. This is in keeping with a previous study, in which no alteration in nasal microbiota composition was observed after continuous positive airway pressure treatment [41], a process known to improve obstructive sleep apnea.

The Spearman’s correlation analysis of our oral microbial sequencing data demonstrated that at baseline, phyla *Firmicutes* was positively correlated with PSQI score but negatively correlated with TWF, but that *Proteobacteria* were negatively correlated with PSQI score and positively correlated with TWF. Our findings are in agreement with those of a previous study, in which an increase in the gut *Firmicutes* population was observed in mice in response to sleep disruption caused by dark-light cycle alternations mimicking night shift work schedules [50]. Furthermore, a higher abundance of *Firmicutes* and a lower abundance of *Proteobacteria* was observed in a mouse model of intermittent hypoxia, which mimics obstructive sleep apnea [51]. Additionally, in a previous study on the association between PSQI and gut microbiota, good sleep quality was found to be positively correlated with *Proteobacteria* abundance [52]. 

At the genus level, we also found a significant correlation between PSQI score and predominant oral microbiota. More specifically, PSQI score was positively correlated with *Veillonella* and *Streptococcus* and negatively correlated with *Neisseria*. These findings are in line with those of a clinical study that reported greater abundances of *Streptococcus* and *Veillonella* in the nasal microbial population of patients suffering from severe obstructive sleep apnea [41]. Interestingly, whereas a predominance of *Neisseria* indicates healthy periodontal conditions, an abundance of *Veillonella* signifies poor periodontal conditions [53]. As mentioned earlier, pathological oral conditions have a deleterious effect on cardiac function, either directly or indirectly through the ANS [11,12], and accumulating evidence indicates that cardiac dysfunction is correlated with insomnia when the ANS is impaired [7,8,9]. Our results regarding oral microbiomes at the genus level are consistent with those observed at the phylum level, because *Veillonella* and *Streptococcus* belong to the phylum *Firmicutes* [54] and *Neisseria* belongs to the phylum *Proteobacteria* [55]. In addition to PSQI, *Veillonella* also showed a positive correlation with CIE-a* and diastolic BP in the present study. Since CIE-a* indicates redness of the tongue body and yin-deficient patients are more likely to have a reddish tongue [19], it is conceivable that *Veillonella* is an indicator of insomnia, particularly in the presence of a yin-deficient pattern. In addition to the dominant oral microbial genera mentioned above, *Peptococcus*, *Fusobacterium*, and *Porphyromonas* all showed negative correlations with PSQI; *Peptococcus* and *Fusobacterium* demonstrated a negative correlation with WMT; and *Porphyromonas* exhibited negative and positive correlations with PR and TST, respectively. Taken together and considering the fact that more severe insomnia is associated with lower blood pressure and a higher pulse rate due to autonomic imbalance [56], increased abundances of *Peptococcus*, *Fusobacterium*, and *Porphyromonas* may indicate sleep improvements.

Current evidence indicates that autonomic nerve dysfunction is a potential mechanism of insomnia, and therefore, insomnia patients tend to have increased sympathetic and reduced parasympathetic tones [57]. In HRV, high-frequency power (HF) is attributed to parasympathetic nerve activity, while low-frequency power (LF) indicates functions related to sympathetic and vagal nerves. In order to minimize the effects of very low frequency (VLF) on HF or LF, we used normalized HF (HFn) and normalized LF (LFn) to focus on maintaining balance between sympathetic and parasympathetic nerves; moreover, LFn more correctly represents sympathetic nerve activity than LF [58,59]. In our study, *Lautropia* showed a positive correlation with HFn and negative correlations with LFn and ISI. Thus, the higher the abundance of *Lautropia*, the higher the activity of the parasympathetic nervous system as compared with the sympathetic nervous system. *Lautropia* belongs to the phylum *Proteobacteria* [60], the abundance of which was negatively correlated with PSQI in the present study. In contrast, *Selenomonas* and *Leptotrichia* were negatively correlated with HFn and positively correlated with LFn, which suggests these two oral microbiotas are more related to the SNS than the parasympathetic nervous system and that *Lautropia* is associated with good sleep quality, whereas *Selenomonas* and *Leptotrichia* are related to sleep disorders.

In order to understand relationships between oral microbiota and sleep and associated factors, we dichotomized all subjects by orotype using oral microbial features regardless of gender, age, or BMI. At baseline, the PSQI and pulse rate of orotype 1 were significantly greater than those of orotype 2. In addition, the BDI and ISI of orotype 1 were non-significantly higher than those of orotype 2. Accordingly, patients of orotype 1 tended to have poorer mood and sleep quality and a higher pulse rate than those of orotype 2. This is in agreement with a previous study that reported that insomnia symptoms were positively related to depression and pulse rate [56], and is similar to the differences observed between the HYD and NHYD groups in the present study. We also found that the abundances of *Prevotella* and *Veillonella* in orotype 1 were significantly greater than in orotype 2, but the abundance of *Neisseria* in orotype 2 was significantly greater than in orotype 1. These findings are in keeping with a previous report (mentioned above) that showed that *Prevotella* and *Veillonella* in oral microbiomes are correlated with periodontal disease, whereas *Neisseria* is correlated with healthy periodontal conditions [53]. After treatment with CWBSD, ISI and PSQI scores declined in both orotypes. In particular, the reduction in PSQI was significantly greater for orotype 1 than orotype 2, which is similar to the responses of the HYD and NHYD groups. This is an interesting observation considering that both orotype 1 and the HYD group had higher *Veillonella* abundances. However, no significant intra- or inter-orotype differences were observed between actigraphs, tongue diagnostic features, cardiac function, autonomic nerve function, or oral microbial populations in subjects in response to CWBSD treatment.

To predict CWBSD response in terms of sleep quality, we developed an equation based on oral microbial patterns of five types of bacteria, namely, *Paludibacter*, *Selenomonas*, *Peptococcus*, *Peptostreptococcus*, and *Gemella*. Previous reports show oral halitosis is associated with higher abundances of *Selenomonas* and *Peptococcus* [61], and that *Paludibacter* is associated with dental health [62], whereas patients with severe obstructive sleep apnea have higher oral abundances of *Gemella* [41]. On the other hand, *Peptostreptococcus* in the gut has a beneficial effect on the intestinal epithelium and alleviates inflammation by producing indoleacrylic acid [63]. Based on the functions of these microbiotas, it is conceivable that CWBSD treatment has a greater impact on insomnia patients with more disease-related microbial populations. Sensitivity and specificity are traditionally used to assess the accuracy of diagnostic tests [64] and these two measures were relatively high for our prediction equation. However, cross-sectional or cohort studies are required to predict the accuracy of this equation [65]. Additionally, most participants in the present study were women with ages ranging from 40 to 50 years and our equation does not include other parameters, such as age, sex, PSQI, actigraphy features, tongue characteristics, etc., which cautions against its application to all insomnia patients.

To the best of our knowledge, this is the first study to investigate the effect of CWBSD on insomnia and associated factors such as cardiac function, autonomic nerve function, tongue features, and oral microbiota. However, the sample size was small and no placebo was used. Furthermore, it has been suggested that oral microbial communities depend on gender, age, and smoking status [66], and thus, these factors are likely to act as confounders. Therefore, we recommend that further larger-scale studies that include a placebo control and adjustment for confounders should be conducted to support our findings.

## 5. Conclusions

In conclusion, the present study reveals CWBSD treatment improves sleep quality in insomnia patients, but that its effects are more pronounced in HYD patients than NHYD patients. Furthermore, CWBSD-induced improvements in sleep were associated with an increase in coating at the root of the tongue, which is where the heart opens according to the theory of traditional medicine. Furthermore, at baseline, the HYD group had a higher oral population of *Veillonella* than the NHYD group. However, its abundance was not changed by CWBSD treatment in either group. Subjects were also divided by orotype, and orotype 1 showed a significantly higher abundance of *Prevotella* and *Veillonella* than orotype 2, whereas orotype 2 exhibited a significantly higher abundance of *Neisseria* than 1. These findings suggest that *Veillonella* may be a diagnostic indicator of CWBSD responsiveness in insomnia patients. We believe that the findings of the current study demonstrate that CWBSD provides a developmental basis for a personalized medicine that improves primary insomnia.

## Figures and Tables

**Table 1:** Composition of CWBSD.

Botanical Name	Amount (mg)
*Rehmanniae radix*	500
*Coptidis rhizoma*	250
*Angelicae gigantis radix*	125
*Asparagi radix*	125
*Schisandrae fructus*	125
*Biotae semen*	125
*Ziziphi spinosae semen*	125
*Liriopis tuber*	125
*Ginseng radix*	62.5
*Hoelen*	62.5
*Platycodi radix*	62.5
*Polygalae radix*	62.5
*Scrophulariae radix*	62.5
*Salviae miltiorrhizae radix*	62.5

**Table 2:** Baseline demographic and clinical characteristics.

Variables	HYD (n = 17)	NHYD (n = 39)	*p* Value *
Gender, No. (male/female)	2/15	7/32	0.707 ^†^
Drinking, No. (yes/no)	5/12	16/23	0.409 ^††^
Smoking, No. (yes/no)	2/15	2/37	0.577 ^†^
Caffeine, No. (yes/no)	10/7	22/17	0.867 ^††^
Age (years)	48.41 ± 9.49	51.38 ± 9.14	0.391
BMI (kg/m^2^)	23.22 ± 2.59	22.47 ± 2.60	0.325
BDI	15.65 ± 6.12	10.72 ± 5.80	0.007
ISI	20.94 ± 4.78	17.82 ± 5.00	0.034
PSQI	14.35 ± 2.94	12.56 ± 3.77	0.088
Systolic BP (mmHg)	121.41 ± 15.80	122.97 ± 12.73	0.696
Diastolic BP (mmHg)	71.94 ± 11.32	73.69 ± 8.45	0.524
Pulse rate (/min)	79.71 ± 11.08	74.82 ± 9.23	0.092

Data presented as mean ± standard deviation. * *p* value: obtained from independent two sample *t*-test. ^†^ *p* value: obtained from Fisher exact test. ^††^ *p* value: obtained from Pearson chi-square test. HYD: heart yin deficiency; HYD: non-heart yin deficiency; BMI: body mass index; BDI: Beck Depression Inventory; ISI: Insomnia Severity Index; PSQI: Pittsburgh Sleep Quality Index; BP: blood pressure.

**Table 3:** Baseline demographic and clinical characteristics of each orotype.

Characteristics	Orotype 1 (n = 20)	Orotype 2 (n = 26)	Between Group *p* Value *
Gender, No. (male/female)	3/17	4/22	1.000 ^†^
Drinking, No. (yes/no)	8/12	8/18	0.515 ^††^
Smoking, No. (yes/no)	3/17	0/26	0.075 ^†^
Caffeine, No. (yes/no)	13/7	15/11	0.615 ^††^
Age (years)	48.50 ± 11.95	51.08 ± 7.56	0.406
BMI (kg/m^2^)	22.92 ± 2.64	22.97 ± 2.07	0.945
BDI	14.10 ± 7.35	11.65 ± 5.71	0.227
ISI	19.55 ± 5.12	18.12 ± 5.71	0.382
PSQI	14.30 ± 3.26	11.69 ± 3.77	0.018 *
Systolic BP (mmHg)	122.40 ± 15.00	116.19 ± 12.62	0.135
Diastolic BP (mmHg)	74.60 ± 11.74	69.50 ± 8.33	0.092
Pulse rate (/min)	77.50 ± 6.75	72.23 ± 9.77	0.036 *

Data presented as mean ± standard deviation. * *p* value: obtained from independent two sample *t*-test. ^†^ *p* value: obtained from Fisher exact test. ^††^ *p* value: obtained from Pearson Chi-square test. HYD: heart yin deficiency; NHYD: non-heart yin deficiency; BMI: body mass index; BDI: Beck Depression Inventory; ISI: Insomnia Severity Index; PSQI: Pittsburgh Sleep Quality Index; BP: blood pressure.

## Data Availability

The data supporting this study are available on request from the corresponding author.

## Supplementary Materials

The following are available online at https://www.mdpi.com/article/10.3390/jpm11050325/s1, Figure S1: Diversity of oral microbiota before and after CWBSD administration, Table S1: Effects of CWBSD on tongue features, Table S2: Effects of CWBSD on cardiac and autonomic nerve function, Table S3: Effects of CWBSD on complete blood count, Table S4: Effects of CWBSD on tongue features, cardiac function, and autonomic nerve function of each orotype.

## Abbreviations

CWBSD: Cheonwangbosim-dan; HYD: heart-yin deficiency; NHYD: non heart-yin deficiency; ANS: autonomic nervous system; TKM: traditional Korean medicine; TDS: tongue diagnosis system; GMP: good manufacturing practice; PSQI: Pittsburgh Sleep Quality Index; ISI: Insomnia Severity Index; BDI: Beck Depression Index; PIT-Insomnia: Pattern Identification Tool-Insomnia; HRV: heart rate variability; WTCI: Winkel Tongue Coating Index; QIIME: Quantitative Insights Into Microbial Ecology; LEfSe: linear discriminant analysis effect size; LDA: linear discriminant analysis; WBC: white blood cell; RBC: red blood cell; Hb: hemoglobin; Hct: hematocrit; PLT: platelet; NEU: neutrophil; LYM: lymphocyte; TBT: total bed time; TST: total sleep time; HF: high-frequency power; LF: low-frequency power; VLF: very low frequency; HFn: normalized HF; LFn: normalized LF.
