# Supplementary material for: The Herbal Formula CWBSD Improves Sleep Quality Dependent on Oral Microbial Type and Tongue Diagnostic Features in Insomnia
Source: J Pers Med. 2021 Apr 21;11(5):325. doi: 10.3390/jpm11050325 (PMC8143156; doi:10.3390/jpm11050325)
Supplement: Supplementary file 1 [file jpm-11-00325-s001.zip › jpm-1167034-supplementary.pdf]

The herbal formula CWBSD improves sleep quality dependent on oral microbial type and tongue diagnostic features in insomnia

Supplementary information

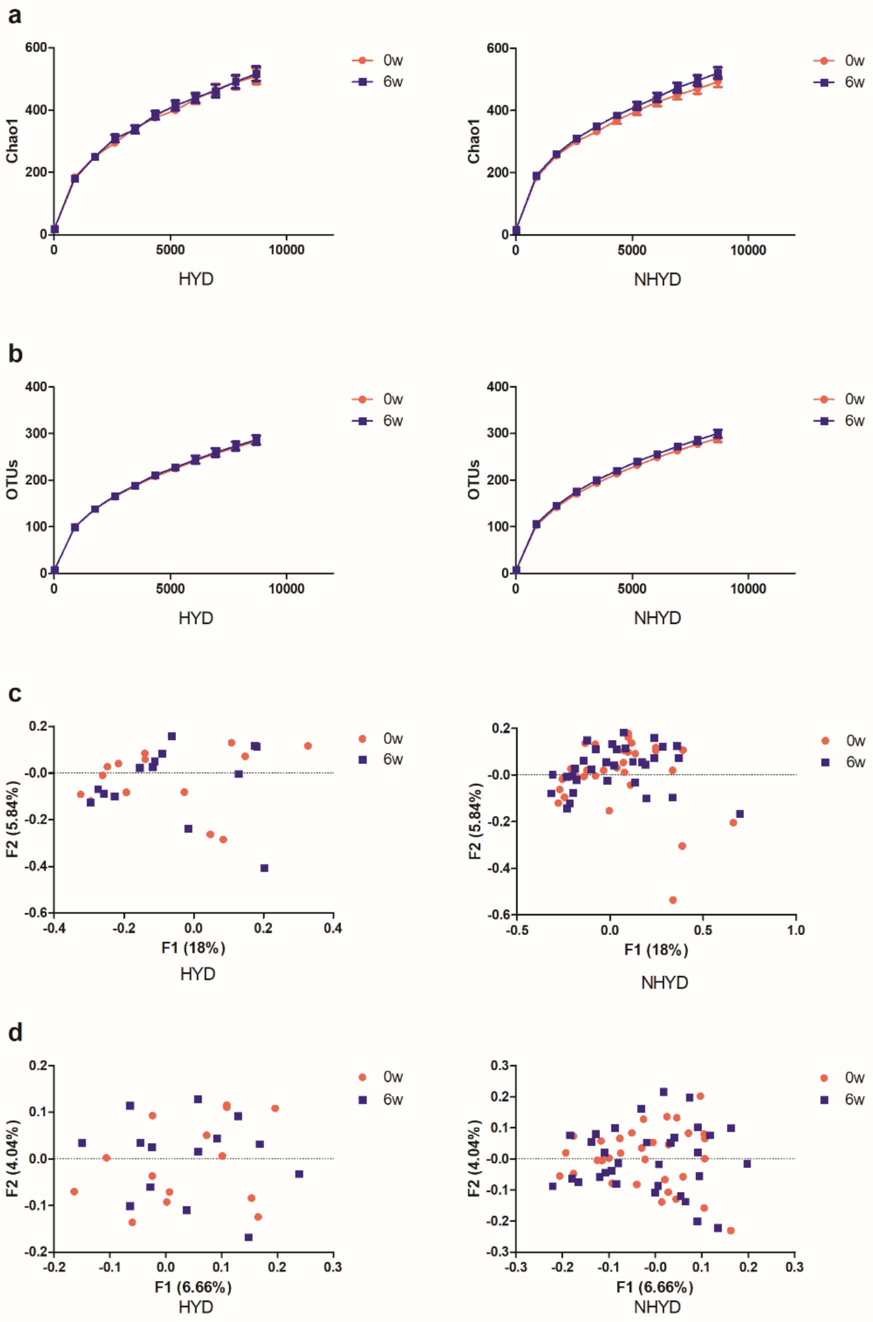
a
Chao1
0w
6w
HYD
NHYD
b
OTUs
0w
6w
HYD
NHYD
c
F2 (5.84%)
F1 (18%)
0w
6w
HYD
NHYD
d
F2 (4.04%)
F1 (6.66%)
0w
6w
HYD
NHYD

Figure S1. Diversity of oral microbiota before and after CWBSD administration

Alpha diversity of bacterial species in the HYD and NHYD groups as indicated by a) Chao1 indices and b) OTUs. Beta diversity of bacterial species as indicated by c) weighted and d) unweighted Unifrac distances.

CWBSD: Cheonwangbosim-Dan.

Table S1. Effects of CWBSD on tongue features

Variables	HYD (n=15)			NHYD (n=37)			Between group P value§
	Week 0	Week 6	within††	Week 0	Week 6	within††	
R_TB	164.69 ± 8.29	166.65 ± 7.47	0.326	167.72 ± 7.16	166.12 ± 7.13	0.234	0.146
G_TB	103.40 ± 7.89	106.25 ± 7.05	0.165	106.57 ± 6.42	104.84 ± 7.98	0.219	0.073
B_TB	97.08 ± 7.54	101.31 ± 6.62	0.094	100.08 ± 8.31	98.21 ± 7.93	0.212	0.031*
L_TB	50.22 ± 2.99	51.25 ± 2.59	0.174	51.43 ± 2.44	50.78 ± 2.76	0.206	0.073
a_TB	23.87 ± 1.73	23.56 ± 1.91	0.565	23.66 ± 2.01	23.77 ± 2.53	0.771	0.545
b_TB	13.89 ± 2.35	12.85 ± 3.01	0.130	13.86 ± 2.73	14.03 ± 2.81	0.686	0.125
R_TC	153.94 ± 15.50	151.40 ± 16.88	0.516	153.85 ± 21.71	157.74 ± 16.96	0.314	0.325
G_TC	117.88 ± 16.02	114.81 ± 16.92	0.446	119.38 ± 21.78	122.91 ± 17.73	0.340	0.298
B_TC	104.18 ± 18.05	102.05 ± 18.94	0.677	106.26 ± 24.07	110.46 ± 21.20	0.302	0.374
L_TC	52.67 ± 6.22	51.54 ± 6.71	0.473	53.04 ± 8.61	54.52 ± 6.89	0.320	0.303
a_TC	12.16 ± 1.05	12.59 ± 0.75	0.085	11.59 ± 1.24	11.75 ± 1.26	0.420	0.412
b_TC	13.04 ± 3.42	12.69 ± 3.47	0.687	12.46 ± 3.80	12.08 ± 3.92	0.524	0.972
Pixel_Tongue	31614.67 ± 5016.68	29889.53 ± 5112.64	0.026*	33587.27 ± 6335.98	32549.65 ± 5615.03	0.073	0.490
Pixel_TC	4199.80 ± 3218.68	4343.80 ± 2984.74	0.844	5223.03 ± 3866.47	4471.68 ± 3464.53	0.267	0.438
PTC	12.91 ± 9.23	14.51 ± 9.63	0.492	15.73 ± 12.33	13.89 ± 10.89	0.362	0.324

Data presented as mean±standard deviation. † P-value by the paired t-test; § P-value by the Mann-Whitney test or the independent t-test; * $p<0.05$.

R_TB: Mean red value of pixels in tongue body area; G_TB: Mean green value of pixels in tongue body area; B_TB: Mean blue value

of pixels in tongue body area; L_TB: Mean CIE-lightness value of pixels in tongue body area; a_TB: Mean CIE-red saturation value of pixels in tongue body area; b_TB: Mean CIE-yellow saturation value of pixels in tongue body area; R_TC: Mean red value of pixels in tongue coating area; G_TC: Mean green value of pixels in tongue coating area; B_TC: Mean blue value of pixels in tongue coating area; L_TC: Mean CIE-lightness value of pixels in tongue coating area; a_TC: Mean CIE-red saturation value of pixels in tongue coating area; b_TC: Mean CIE-yellow saturation value of pixels in tongue coating area; Pixel_TC: Count of Pixel in tongue coating area; PTC: percentage of tongue coating area to whole tongue area; CWBSD: Cheonwangbosim-Dan.

Table S2. Effects of CWBSD on cardiac and autonomic nerve function

Variables	HYD (n=15)			NHYD (n=37)			Between group P value§
	Week 0	Week 6	within††	Week 0	Week 6	within††	
Mean HRT (bpm)	71.87 ± 6.59	69.27 ± 12.62	0.431	70.08 ± 8.21	68.43 ± 7.34	0.107	0.711
Heart stability	92.53 ± 16.40	92.20 ± 19.50	0.934	98.16 ± 18.11	98.08 ± 18.72	0.974	0.957
HFn (n.u.)	41.62 ± 20.64	46.54 ± 27.66	0.417	45.06 ± 24.30	47.57 ± 19.87	0.507	0.730
LFn (n.u.)	58.38 ± 20.64	53.46 ± 27.66	0.417	54.94 ± 24.30	52.43 ± 19.87	0.507	0.730
VLF (ms2)	523.34 ± 585.35	817.39 ± 1312.96	0.280	601.91 ± 1000.27	621.79 ± 606.01	0.916	0.801
LF/VF ratio	2.13 ± 1.78	3.66 ± 7.02	0.336	2.69 ± 3.36	1.83 ± 2.22	0.081	0.413

Data presented as mean±standard deviation. † P-value by the Wilcoxon signed rank test; †† P-value by the paired t-test; § P-value by the Mann-Whitney test or the independent t-test; * $p<0.05$.

Mean HRT: mean heart rate; HFn: High Frequency power in normalized units; LFn: Low Frequency power in normalized units; VLF: Very Low Frequency; LF/VF ratio: Low Frequency/ High Frequency ratio; CWBSD: Cheonwangbosim-Dan.

.

Table S3. Effects of CWBSD on complete blood count

Variables	HYD (n=15)			NHYD (n=37)			Between group P value§
	Week 0	Week 6	within††	Week 0	Week 6	within††	
WBC ($\times 10^3/\mu\ell$)	6.50 ± 2.33	6.38 ± 1.88	0.737	5.79 ± 1.14	6.20 ± 1.71	0.061	0.192
RBC (×106/$\mu\ell$)	4.10 ± 0.33	4.16 ± 0.36	0.275	4.34 ± 0.43	4.40 ± 0.41	0.123	0.975
Hb (g/dL)	12.66 ± 0.83	12.88 ± 0.86	0.194	13.22 ± 1.69	13.38 ± 1.66	0.089	0.718
Hct (%)	37.67 ± 2.37	38.46 ± 2.40	0.087	39.34 ± 4.15	39.85 ± 3.93	0.094	0.607
Platelet ($\times 10^3/\mu\ell$)	257.33 ± 58.65	257.87 ± 47.90	0.926	249.59 ± 50.78	257.08 ± 48.94	0.100	0.379
Neutrophil(%)	56.28 ± 11.65	56.05 ± 9.95	0.894	51.01 ± 6.24	52.80 ± 8.34	0.128	0.343
Lymphocyte(%)	34.27 ± 9.74	34.42 ± 8.45	0.923	38.21 ± 5.90	36.79 ± 7.86	0.175	0.409

Data presented as mean±standard deviation. † P-value by the paired t-test; § P-value by the independent t-test; * $p<0.05$.

WBC: white blood cell; RBC: red blood cell; Hb: hemoglobin; Hct: hematocrit; CWBSD: Cheonwangbosim-Dan.

Table S4. Effects of CWBSD on tongue features, cardiac function and autonomic nerve function of each orotype

Variables	Orotype 1 (n=20)			Orotype 2 (n=26)			Between group P value§
	Week 0	Week 6	within††	Week 0	Week 6	within††	
L_TB	50.78 ± 2.76	51.49 ± 2.50	0.325	51.65 ± 2.39	50.96 ± 2.64	0.250	0.130
a_TB	23.65 ± 1.75	23.66 ± 2.20	0.978	23.63 ± 2.11	23.52 ± 2.65	0.806	0.861
L_TC	53.27 ± 8.19	53.22 ± 6.93	0.980	52.64 ± 8.17	55.27 ± 5.92	0.094	0.281
b_TC	12.08 ± 3.20	12.28 ± 3.44	0.847	12.73 ± 3.99	12.18 ± 4.04	0.322	0.493
PTC	16.84 ± 13.96	14.76 ± 11.16	0.505	14.15 ± 9.84	15.19 ± 10.52	0.579	0.365
Systolic BP (mmHg)	122.40 ± 15.00	120.85 ± 14.54	0.696	116.19 ± 12.62	120.19 ± 14.43	0.334	0.078
Diastolic BP (mmHg)	74.60 ± 11.74	73.55 ± 12.72	0.748	69.50 ± 8.33	73.77 ± 9.16	0.110	0.053
Pulse rate (/min)	77.50 ± 6.75	76.25 ± 9.84	0.666	72.23 ± 9.77	70.81 ± 6.62	0.502	0.954
Mean HRT (bpm)	73.90 ± 7.03	70.10 ± 9.99	0.225	68.27 ± 7.87	68.35 ± 8.35	0.974	0.129
Heart stability	89.00 ± 15.11	91.05 ± 16.00	0.699	100.65 ± 18.33	97.58 ± 17.66	0.584	0.205
HFn (n.u.)	39.06 ± 21.16	45.45 ± 24.67	0.380	47.45 ± 23.39	47.91 ± 19.70	0.942	0.389
LFn (n.u.)	60.94 ± 21.16	54.55 ± 24.67	0.380	52.55 ± 23.40	52.10 ± 19.70	0.942	0.389
VLF (ms2)	434.27 ± 544.37	805.70 ± 1174.55	0.246	690.02 ± 1148.62	598.71 ± 606.51	0.732	0.186
LF/HF ratio	2.88 ± 3.26	3.26 ± 6.15	0.818	2.13 ± 2.58	1.67 ± 1.87	0.512	0.570

Data presented as mean±standard deviation. † P-value by the paired t-test; § P-value by the independent t-test; * p<0.05.

L_TB: CIE-lightness of tongue body; a_TB: CIE-red saturation of tongue body; L_TC: CIE-lightness of tongue coating; b_TC: CIE-yellow saturation of tongue coating; PTC: percentage of tongue coating area to whole tongue area; BP: Blood Pressure; Mean HRT: mean heart rate; HFn: High Frequency power in normalized units; LFn: Low Frequency power in normalized units; VLF: Very Low Frequency; LF/HF ratio: Low Frequency/ High Frequency ratio; CWBSD: Cheonwangbosim-Dan.

.
